# The Enhancement of the Perception of Saltiness by Odorants Selected from Chinese Douchi in Salt Solution

**DOI:** 10.3390/foods11152260

**Published:** 2022-07-28

**Authors:** Ya Gao, Wenqian Wang, Huiying Zhang, Haitao Chen, Shuqi Wang, Baoguo Sun

**Affiliations:** Beijing Key Laboratory of Flavor Chemistry, Beijing Technology and Business University, Beijing 100048, China; gy24717@163.com (Y.G.); wwwenqian1998@163.com (W.W.); chenht@th.btbu.edu.cn (H.C.); wsq930312@163.com (S.W.); sunbg@btbu.edu.cn (B.S.)

**Keywords:** odor-induced taste enhancement, Douchi, odorant, gas chromatography-olfactometry/associated taste, saltiness perception

## Abstract

Douchi is a traditional fermented soya bean product that is popular for its smelled saltiness and unique flavor. In order to look for the relationship between smelled saltiness of volatiles and their saltiness-enhancing properties, gas chromatography-olfactometry/associated taste was used to select odorants associated with saltiness in Yongchuan Douchi. The enhancement effects of saltiness intensity by selected odorants were further verified by sensory evaluation analyses of 2-alternative forced-choice and odor-induced saltiness enhancement in a follow-up study. A total of 14 odorant compounds were selected for their odor-associated saltiness perception. The compounds of 2-ethyl-3,5-dimethyl pyrazine, 2,5-dimethyl pyrazine, dimethyl trisulfide, 3-(methylthio) propanol and 3-(methylthio) propanal could significantly enhance saltiness perception in salt solution. Among them, 2-ethyl-3,5-dimethyl pyrazine was reported for the first time to be able to improve the salty taste. The study suggested that salty food is an ideal material for selecting saltiness-enhancing odorants, which could provide more direct theoretical support for salt reduction in the food industry.

## 1. Introduction

The overconsumption of salt in the diet has undesirable effects on health, for example, increasing cardiovascular risk and contributing to other diseases, such as stomach cancer and chronic nephropathy [1,2,3,4]. Consequently, reducing the salt added to food is very important to improve human nutrition and health [5]. Over the past decade, researchers have explored different ways to reduce salt content in foods while maintaining their sensory acceptability [6]. Strategies for using inorganic salts such as potassium chloride instead of sodium chloride have been widely used [7,8,9]. However, the undesired metallic and bitter taste associated with these molecules limited their application [10]. Other strategies, such as modification of food texture, changes of NaCl crystal size, addition of taste enhancers [11], were also used to reduce saltiness in food, but they could not fully meet the requirements to lower sodium consumption without compromising on quality and taste [12].

Recently, cross-modal odor-taste interactions, i.e., odor-induced taste enhancement, have been recognized as an effective way to enhance the perception of saltiness in low-salt foods. That is to say, aroma could increase the intensity of taste, especially when there was some logical relationship between them [13]. Over the last decade, the approach was successful in increasing the saltiness perception. Examples included enhancing the saltiness in cheese by adding ham or sardine odors [14], increasing the perception of saltiness in low-salt content solutions by adding anchovy and bacon aromas [15]. However, the aromas of tomatoes and carrots could not help to enhance saltiness intensity [16]. This indicated that the type of odor determined whether the saltiness perception could be enhanced or not. Congruency might play an important role in success of taste enhancement. In other words, the odor-taste pairs should co-occur in foods and be consistent with associations that are commonly experienced. For example, the strawberry odor could enhance the sweetness of sucrose but it could not increase the saltiness intensity of salt solution.

To understand whether it was the perceived aroma or it was some number of the component volatiles that might be capable of enhancing saltiness intensity, several studies were conducted to focus on the contribution of each aroma compound or their combination. Lopez et al. [17] screened key aroma compounds by calculating the odor activity value, and then established an aroma model including odorants of 2-furfurylthiol and 1-(2-furyl) ethanethiol, improving the saltiness perception of low-sodium chicken broth. He et al. [18] used vacuum simultaneous steam distillation and extraction, aroma extract dilution analysis (AEDA), gas chromatography−mass spectrometry (GC-MS) and 2-alternative forced-choice (2-AFC) to determine that 1-octen-3-ol, ethyl caproate, and phenylacetaldehyde were powerful umami enhancers. Stevenson, Prescott and Boakes [19] used the conception of “smelled taste” to evaluate sweetness and sourness of the odorants and based on this idea, gas chromatography-olfactometry/associated taste (GC-O/AT) was considered as a reliable method for selecting odorants that could enhance the taste perception [20]. Using GC-O/AT, Zhou et al. [21] screened the odorant compounds related to saltiness in soy sauce, found 3-(methylthio) propanal, 1-octen-3-ol, and 2,5-dimethyl pyrazine could clearly increase the saltiness perception of 0.3% NaCl solution by the odor-induced saltiness enhancement (OISE) method.

Douchi is a traditional Chinese specialty of fermented bean products, dating back to more than 2000 years. Douchi, soy sauce, sufu and dajiang are known as the four traditional fermented soybean products in China [22]. Among them, the brewing technology of Yongchuan Douchi is a national intangible cultural heritage. It has been frequently used in Chinese dishes, contributing to a uniquely pleasant aroma and savory taste [23]. The aroma-active substances were detected and identified in previous studies [24]. Although the aroma of Douchi could be described as smelling saltiness, it was still unknown whether Douchi aroma or the individual odorant could enhance saltiness perception in salt solutions.

In the present study, the odorant compounds associated with saltiness in Yongchuan Douchi were screened by GC-O/AT, and their effect of increasing saltiness perception in low-salt solutions was further verified using 2-AFC and OISE methods.

## 2. Materials and Methods

### 2.1. Materials

Yongchuan Douchi was purchased from Waizumu Food Co., Ltd. (Chongqing, China). After being delivered to the laboratory, the Douchi sample was stored at −20 °C until use for volatile compounds extraction.

### 2.2. Chemicals

Dichloromethane and anhydrous sodium sulfate were obtained from Sinopharm Chemical Reagent Co., Ltd. (Beijing, China). Before using, dichloromethane was redistilled to remove impurities. 2-Methyl-3-heptanone (≥95%) and n-alkanes (C6−C30, ≥99) were obtained from Sigma-Aldrich (Shanghai, China). Standard odorants for identification were obtained either from J&K Scientific Co., Ltd. (Beijing, China) or from Aladdin Biochemical Technology Co., Ltd. (Shanghai, China). For sensory evaluation, eight food-grade odorant compounds, including 2-ethyl-3,5-dimethylpyrazine, 2,5-dimethyl pyrazine, 2,6-dimethyl pyrazine, 2-ethyl-5(6)-methyl pyrazine, dimethyl trisulfide, 3-(methylthio) propanol, tetramethyl pyrazine, 3-(methylthio) propanal were obtained from Meixin Chemical Co., Ltd. (Shanghai, China). Purified drinking water was purchased from Wahaha Group Co., Ltd. (Hangzhou, China). Food-grade sodium chloride was purchased from China National Salt Industry Group Co., Ltd. (Beijing, China).

### 2.3. Solvent-Assisted Flavor Extraction (SAFE)

Douchi (50 g) was ground into powder and mixed with 100 mL of dichloromethane and 100 µL of 2-methyl-3-hepanone (1.632 µg/µL, internal standard). The mixture was shaken at room temperature for 2 h and then filtered. The residue was further extracted twice with dichloromethane (total 300 mL). Subsequently, the extracts combination was introduced into the SAFE extractor (Shenxian Jingxing Glassware Co., Ltd., Liaocheng, China) at 45 °C to obtain the volatile compounds [19]. Then the distillate was dehydrated with anhydrous sodium sulfate. After storing at −20 °C overnight, the distillate was concentrated to 1.5 mL using a Vigreux column (50 cm × 1 cm i.d.) and filtered through a 0.22 μm organic membrane. Finally, the concentrated distillate was stored at −40 °C until further analyses [21]. Extractions were performed in triplicate.

### 2.4. Gas Chromatography–Mass Spectrometry (GC–MS) Analysis

GC–MS analysis was performed using the Thermo Fisher Trace 1310 gas chromatograph (Thermo Fisher Scientific, Waltham, MA, USA), with a mass-selective detector (Thermo Fisher Scientific, Waltham, MA, USA). Two capillary columns differing in polarities (TG-WAX and TG-5 capillary columns, 30 m × 0.25 mm × 0.25 µm; Thermo Fisher Scientific, Waltham, MA, USA) were employed to separate volatiles. Helium as a carrier gas was passed through the column at 1.0 mL/min. The inlet was conducted in splitless mode at 250 °C and the injection volume was programmed as 1 μL. The analytical conditions referred to reports of Wang et al. [24]. For the TG-WAX capillary column, the temperature of the oven was maintained at 40 °C for 3 min, then increased to 120 °C at 5 °C/min and held for 4 min, increased to 200 °C at 5 °C/min, finally increased to 240 °C at 5 °C/min and held for 8 min. For the TG-5 column, the oven temperature was maintained at 40 °C for 1 min initially, then increased at 3 °C/min to 100 °C and held for 3 min, increased at 5 °C/min to 280 °C for 3 min, finally increased to 300 °C at 10 °C/min. The mass spectrometry detector conditions were as follows: ion source temperature, 250 °C; transfer line temperature, 240 °C; ionization energy, 70 eV; mass range, 45 to 450 *m/z*. Most volatiles were identified based on their odor characteristics, retention indexes (RIs, on polar and non-polar gas chromatography columns), and mass spectrometry with standard substances [25]. For volatile compounds without references, they were tentatively identified by comparing their RIs with those of known pattern in the NIST 14.0 (National Institute of Standards and Technology, Gaithersburg, MD, USA) database.

### 2.5. Gas Chromatography-Olfactometry/Associated Taste (GC-O/AT) Analysis

GC-O/AT analysis was performed on a Thermo Fisher Trace 1310 gas chromatograph and a sniffing port (ODP3; Gerstel, Mülheim an der Ruhr, Germany). The flow rate of helium was 1.6 mL/min. Each extract (1 μL) was injected in a TG-Wax column. The analytical conditions for the GC were identical to those of GC-MS described in Section 2.4. To avoid high boiling point compounds condensing during the smelling experiment, the olfactory port was maintained at 250 °C using a thermostat. A humidifier was used to prevent the nasal mucosa from drying out [21].

The distillate was analyzed by ten experienced panelists from Beijing Key Laboratory of Flavor Chemistry in Beijing Technology and Business University. They were trained to be familiar with the GC-O/AT procedure in advance. The panelists were requested not to eat or drink one hour before the test. The procedure consisted of two steps. In the process of routine GC-O analysis, the evaluators used their noses as odor detector and recorded the odor description, retention time, and aroma intensity. During the second run, namely the GC-O/AT operation, five taste descriptors: “sweet,” “salty,” “sour,” “umami,” or “bitter” were used to describe the odorants instead of an aroma description. If it was difficult to define the taste attribute of the stimulus immediately, panelists could make a free description first. After the experiment, the group would discuss. If there was any dispute, supplementary tests could be carried out after the GC-O/AT test. Each test was performed in duplicate. Data processing was carried out using the detection frequency (DF) method [26].

### 2.6. Sensory Evaluation

The sensory analysis was performed according to the approaches mentioned by Inoue et al. [27] and He et al. [18] with a few modifications. Twelve trained panelists (4 males and 8 females) ranged in ages from 23 to 29 years were recruited from the Beijing Key Laboratory of Flavor Chemistry. All panelists provided their written informed consent before they took part in the experiments. In this study, the panelists were trained using differential tests. The panelists were required to sip and spit out three NaCl solutions (0.3%, 0.5% and 0.8%) every 10 s. A rinse of mouth was enforced between assessments to avoid the carry-over effect. After evaluating the three samples, they were asked to rank the intensity of saltiness from weak to strong. Then, panelists were requested to assess the taste of aqueous solutions of the salty-enhancing compound previously reported, 2,5-dimethyl pyrazine (1708 ppb), in salty solution (0.3% NaCl) with and without a nose clip [21,28]. Panelists could recognize the different intensities of the salty taste after training.

The differences in saltiness intensity between the salty solutions with and without odorants were compared using the paired comparison method (ISO 5495:2005). Three concentration gradients were prepared for each odorant. The minimum detection concentration (MDC) was determined by the triangle test. In the test, each panelist was offered three cups of sample (10 mL), one cup of water without odorant and two cups of water containing odorant at 0.1−10 ppb with a water bath at 50 °C. The panelists sipped and spat out the solution and chose a sample that was different from the other two. The lowest concentration of odor detected by two-thirds of the panelists was determined as the middle concentration (MC). Subsequently, the range was narrowed to further screen each odor concentration, with MC as the center; a total of 5 concentrations was selected for testing, and the lowest odor concentration detected by one-third of the panelists was defined as the MDC. Each odorant was prepared at three concentrations of MDC, 0.1 MDC and 10 MDC in 0.3% NaCl solution [27]. One cup of salty solution with odorant (10 mL) and salty solution without odorant (10 mL) were served to the panelists who were instructed to choose the one that they considered saltier. The test was performed in a well-ventilated sensory evaluation room. Each panelist evaluated all samples twice, and a total of 24 evalutions was obtained for each pair. These tests were performed wearing nose clips or not. The experimental results were analyzed by the method of binomial test [18].

The sensory results were further calculated as OISE, which means the difference between the saltiness of a NaCl solution containing odorants and the saltiness of a solution containing only the equal amount of NaCl. The value of OISE was calculated according to Equation (1).

(1) OISE = Si –S0 

where Si means the saltiness of a NaCl solution containing a certain odorant; S0 means the saltiness of a solution containing only the equal amount of NaCl.

Twelve panelists mentioned in the previous test participated in this study. Odorants were added in 0.3% NaCl solution, and their potential abilities to improve the saltiness perception were explored. The sample was kept in an odorless cup. All samples were randomly encoded. Before evaluating samples, panelists were asked to be familiar with reference solutions. The intensity of saltiness was compared with aqueous solutions of NaCl (0.3% NaCl solution was defined as 3 points, and 0.8% NaCl solution was defined as 10 points). During the evaluation, the panelists were required to pour the whole sample into their mouth, rotate the solution in their mouth for 10 s, and evaluate the perceived saltiness intensity after coughing up the sample. All of the samples were assessed in duplicate by each panelist. An interval of 20–30 s between each sample was applied.

### 2.7. Statistical Analysis

The sensory data was analyzed using the paired comparison test (ISO 5495:2005). A *p* level less than 0.05 (the number of correct responses ≥ 17) and 0.01 (the number of correct responses ≥ 19) was defined as being of significant difference. The OISE significance was performed by one-sample *t*-test. A *p* level less than 0.05 was used to compare means and identify samples significantly differed from zero. All statistical analyses were performed using SPSS Statistics 26.0 (SPSS, Inc., Chicago, IL, USA). The figures of OISE were obtained using the OriginPro 2021 (OriginLab Corporation, Northampton, MA, USA).

## 3. Results and Discussion

### 3.1. Selection of Odorants Associated with Salty Taste by GC-O/AT

The volatile components in commercial Yongchuan Douchi were extracted by SAFE and analyzed by GC-MS and GC-O/AT. As shown in Table 1, 45 odorants were identified in Yongchuan Douchi by mass spectrometry, retention index and odor description. To select the odorants associated with taste, the data was processed by detection frequency method. When more than 40% of the panelists described a particular taste property for the same odorant, an association between aroma and taste was established. Among the 45 detected components, 14 odorants were mainly connected with saltiness, 10 with sourness, 16 with sweetness, 4 with bitterness, and 1 with umami.

For saltiness, among the 14 salty taste-associated odorants, there were 7 nitrogenous compounds, 3 sulfur compounds, 2 oxygenated heterocycles, 1 aldehyde and 1 unknown component with “soy sauce-like” note (Table 1). A few panelists also associated these compounds with umami, which might be because umami perception could be enhanced by salty and vice versa. Table 1 suggested that sulfur- and nitrogen-containing compounds were the major classes of aroma compounds and were considered to provide “nutty, roasted, soy sauce-like” notes. These aroma descriptions were considered to be closely related to saltiness in the study of Guichard et al. [29]. Among the sulfur-containing compounds, 3-(methylthio) propanal with “roasted” and “cooked potato” notes was evaluated as “salty” by 85% of panelists. Other two sulfur-containing compounds including 3-(methylthio) propanol and dimethyl trisulfide were found to be associated with saltiness by 55% and 45% panelists, respectively. These compounds were usually detected in samples frequently described as having “salty” or “savory” odor attributes, such as soy sauce [21], dehydrated mushroom [25], and logically associated with saltiness by other authors [29]. Pyrazines associated with saltiness were 2,5-dimethyl pyrazine, 2,6-dimethyl pyrazine, 2-ethyl-3,5-dimethyl pyrazine, tetramethyl pyrazine, 2-ethenyl-6-methyl pyrazine, 2-ethyl-6-methyl pyrazine and 2-ethyl-5-methyl pyrazine. Pyrazines were typical Maillard reaction products and generally reported to have “nutty” and “roasted” properties. These components were discovered in many food systems, including beef products, cocoa products, popcorn, potatoes, soy products, nuts and roasted almonds. They were also considered as microbial metabolites which could be generated by the fermentation process [30]. In our results, 2-pentylfuran and decanal emitted a beany, grassy or fatty odor were also evaluated as “salty” by panelists.

A total of 10 sourness odorants was identified, most of which were organic acids. 3-methylbutanoic and 3-methylvaleric acid were identified with “sweaty” notes and were described as “sour” by 100% of panelists. Other acids were also described as “sour” by more than 60% of panelists. It has been reported that acids were produced by bacteria during pile-fermentation and had limited impact on the pungent flavors of Douchi [30]. In addition, one aldehyde and one ester were found, also described as “sour” by some panelists.

Odorants associated with sweetness formed the biggest group, including alcohols, ketones, aldehydes, furans and phenols mostly. Alcohols associated with sweetness were 3-methyl-1-butanol, benzyl alcohol and phenethyl alcohol, with fruity and floral-related odor attributes. According to several panelists, they were also related to sourness to some extent (15–20%). The ketones associated with sweetness were 2,3-butanedione, trimethyl cyclohexadienyl butanone, 2-hydroxy-1-methylcyclopenten-3-one and 3-methyl-1,2-cyclopentanedione, usually described as “caramel”, “fruity” or “herbal” notes. Among the aldehydes, benzeneacetaldehyde was evaluated as “sweet” by 75% of the panelists. It was reported as the main odorant compound of brown sugar, elicited a sweet note, and could improve the sweetness perception of sweet solutions to a certain degree [31]. Dihydro-5-ethyl-2(3H)-furanone, described as having “caramel” and “sweet” odor properties, was logically related to sweetness with little doubt. As for phenolic components, most of them have an aroma of sweet fruits or mild spices.

There were 4 odorants related to bitterness, including 1 odorant with “spicy” (benzoic acid), 1 odorant with “herbal” (2,3-dihydro-3,5-dihydroxy-6-methyl-4(H)-pyran-4-one) and 2 odorants with “bitterness” notes.

For umami taste, panelists selected one compound, citronellol. This compound was also described as bitter or sour by some panelists. Citronellol was previously reported as one of the main components in wine [32], with a special aroma like fresh roses and a bitter taste.

### 3.2. Evaluation of the Odor-Induced Saltiness Enhancement in Salt Solution

A total of 8 odorants were assessed to determine saltiness-enhancing effects in salt solution. For simplicity, odorants with DF > 30% in other tastes were excluded and considered to be noise. The selected odorant compounds were added to the salt solution. The compounds and their concentrations under three gradients used in this trial were listed in Table 2.

As shown in Table 2, five odorant compounds including 2-ethyl-3,5-dimethyl pyrazine, 2,5-dimethyl pyrazine, dimethyl trisulfide, 3-methylthiopropanol and 3-(methylthio) propanal could enhance the saltiness at very low concentrations without the nose clip. This phenomenon might be due to the simultaneous stimulation of the multisensory integration of the olfactory and taste to produce a taste effect [33]. Studies showed that the taste perception was affected by the sense of smell and the posterior nasal pathway [34]. Retronasal thresholds were usually lower than orthonasal. It was not surprising that some odorant compounds with low concentrations could enhance the intensity of the saltiness perception [35]. So far, whether this cross-modal interaction is a result of cognition, or it is due to certain odorants that might be innately salty has always been a controversial topic. Research has shown that odor-taste perceptual interaction might be influenced by cultural or individual differences. For example, nutmeg, a spice with no obvious flavor, was perceived with distinct sweetness by participants from different regions, and that was because the two groups used different spices in their cooking [36]. In general, these findings suggested that cognitive factors played a key role in the central integration of smell and taste. However, some scholars have raised an intriguing possibility that some odorants were innately sweet or naturally salty, as they could enhance relevant taste perception, even when they were presented at subthreshold levels [37]. In our experiment, the concentration of the selected compounds was very low, and three of them were at subthreshold levels. In odor to verify whether the cognitive factors affected the results, in future experiments, we would consider recruiting panelists with different dietary habits or from different countries to investigate the effects on taste perception. These cross-modal interactions proved the actual taste effects of most selected odorants were consistent with their taste properties obtained in the GC-O/AT process. However, in 0.3% NaCl solution, the other three components namely 2,6-dimethyl pyrazine, 2-ethyl-5(6)-methyl pyrazine and tetramethyl pyrazine related with salty taste in GC-O/AT, could not improve the saltiness perception. This might be related to their complicated taste description in GC-O/AT experiments. The observations indicated that not all odorants smelled as salty could actually induce saltiness enhancement. Sensory analysis was still required to verify the actual odor-induced saltiness enhancement ability of the odorant.

Without nose clip, 2-ethyl-3,5-dimethyl pyrazine could significantly enhance saltiness perception of 0.3% NaCl solution at 2 ppb and 20 ppb (*p* ≤ 0.01). This was the first report describing the effect of 2-ethyl-3,5-dimethyl pyrazine on the saltiness perception of salt solution. With nose clips, salt solutions without odorants were chosen by most panelists. The possible reason was that when 2-ethyl-3,5-dimethyl pyrazine was added in a certain amount, it might have interacted with NaCl molecules.

2,5-Dimethyl pyrazine had a pungent aroma of fried peanuts, and at high concentrations, it was described as astringent. There was a consistency between the aroma attributes and the saltiness, so it was not surprising that 2,5-dimethyl pyrazine had a synergetic effect on salt solution. As shown in Table 2, at 10 ppb and 100 ppb, 2,5-dimethyl pyrazine could obviously increase saltiness perception of 0.3% NaCl solution (*p* ≤ 0.05), and the effective concentration was much lower than that reported in a previous study [21].

3-(Methylthio) propanal (2 ppb) also showed a significant difference (*p* ≤ 0.01) at lower concentrations. 3-(methylthio) propanal could significantly improve the saltiness perception of 0.3% NaCl solution at 2 ppb (*p* ≤ 0.01). With or without nasal clip, 3-(methylthio) propanal could improve the saltiness perception in 0.3% salt solution at 20 ppb.

It was found in Table 2 that saltiness perception could be significantly enhanced by dimethyl trisulfide at 0.25 ppb. However, when the concentration was high, there was no saltiness enhancement effect. Zhou et al. [21] also reported that dimethyl trisulfide (339 ppb) could not significantly increase the saltiness perception of 0.3% NaCl solution. Therefore, dimethyl trisulfide could enhance saltiness perception of low-salt content solutions at a proper concentration.

3-(Methylthio) propanol could significantly enhance the saltiness perception at 250 ppb. Zhao’s [38] research found that 3-(methylthio) propanol had an important contribution to the overall aroma of Pixian broad bean paste. This sulfur-containing compound exhibited cooked potato-like and meaty odor, which had a strong consistency with saltiness perception.

### 3.3. Enhancement of Saltiness Taste by Selected Odorants Using OISE

**Figure 1:**
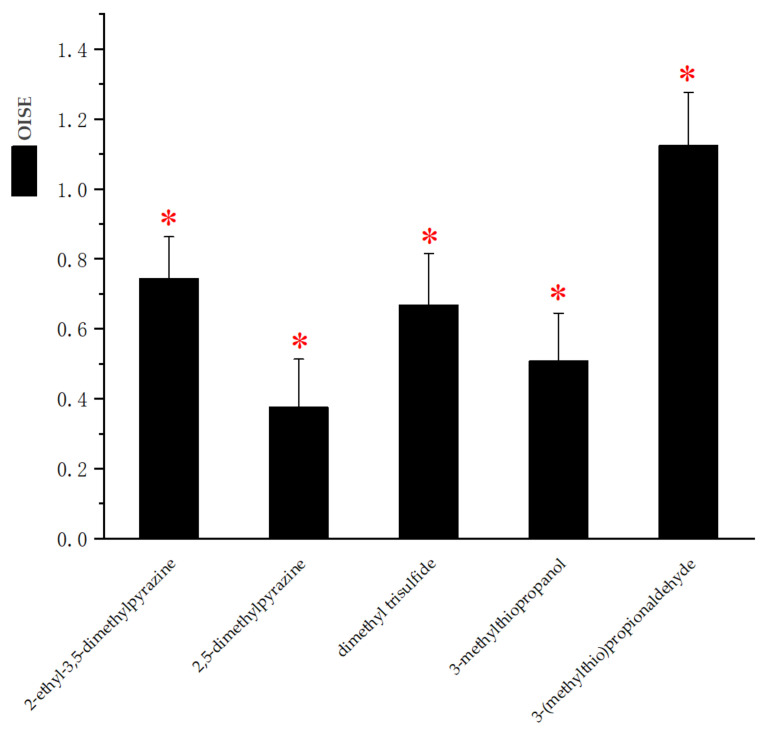
Mean value of odor-induced saltiness enhancement (OISE) in 0.3% NaCl solution with an odorant. Red stars indicated that the mean saltiness intensity was significantly higher than the reference (0.3% NaCl solution): * *p* < 0.05. Error bars represented the standard errors of the mean.

In order to score the saltiness enhancement of solutions with additional aroma, the OISE value of odorants selected by 2-AFC analysis were calculated in this study [39]. Panelists evaluated the saltiness perception intensities of each sample, including NaCl solution alone and the NaCl solution with each odorant during this sensory evaluation. Taking the reference NaCl solution without odorants as reference solution, OISE values were calculated for each odorant and the results are presented in Figure 1. As shown in Figure 1, 2-ethyl-3,5-dimethyl pyrazine, 2,5-dimethyl pyrazine, dimethyl trisulfide, 3-(methylthio) propanol and 3-(methylthio) propanal could all improve the saltiness perception of 0.3% NaCl solution significantly. Among them, 3-(methylthio) propanal had the most obvious effect on enhancing saltiness. The enhancement effect of 2-ethyl-3,5-dimethyl pyrazine was similar to that of dimethyl trisulfide. 2,5-Dimethyl pyrazine and 3-(methylthio) propanol had weak saltiness enhancement effects in NaCl solution. As expected, these results proved that particular odorant compounds at a certain concentration level could enhance the saltiness perception but with different ability.

## 4. Conclusions

A total of 45 odorants were detected by GC-O/AT combined with GC–MS in Yongchuan Douchi and 14 of them were mainly associated with saltiness. The saltiness enhancement abilities of 8 selected odorants were tested in 0.3% NaCl solution. The results proved that 2-ethyl-3,5-dimethyl pyrazine, 2,5-dimethyl pyrazine, dimethyl trisulfide, 3-(methylthio) propanol and 3-(methylthio) propanal could enhance saltiness perception at very low concentrations. Among them, 2-ethyl-3,5-dimethyl pyrazine was reported for the first time to be able to improve the salty taste. This study proved a potential influence of Douchi aroma on salty taste and provided more direct theoretical support for salt reduction in the food industry by cross-modal interaction.

## Figures and Tables

**Table 1:** Odorants identified in Yongchuan Douchi by gas chromatography−mass spectrometry and gas chromatography-olfactometry/associated taste.

No.	Compounds	RI ^a^	Odor Description	Taste Description		Identification Methods ^c^
				Main (DF %) ^b^	Others (DF %)	
Odor descriptors associated with saltiness						
1	2-pentylfuran	1239	green bean	salty (60%)	umami (40%)	RI/MS/O
2	2,5-dimethylpyrazine	1333	nutty	salty (65%)	umami (25%) sour (10%)	RI/MS/O
3	2,6-dimethylpyrazine	1326	nutty	salty (65%)	umami (20%) sour (15%)	RI/MS/O
4	dimethyl trisulfide	1386	leather-like, pickle-like	salty (45%)	bitter (30%) sour (25%)	RI/MS/O
5	2-ethyl-6-methylpyrazine	1394	roasted	salty (40%)	umami (35%) sour (25%)	RI/MS/O
6	2-ethyl-5-methyl-pyrazine	1399	celery-like, roasted	salty (40%)	umami (25%) sweet (20%) bitter (15%)	RI/MS/O
7	3-(methylthio)propanal	1462	roasted, cooked potato	salty (85%)	umami (15%)	RI/MS/O
8	2-ethyl-3,5-dimethylpyrazine	1452	roasted, cacao-like	salty (80%)	umami (20%)	RI/MS/O
9	tetramethyl pyrazine	1476	roasted, cacao-like	salty (70%)	umami (25%) bitter (5%)	RI/MS/O
10	2-ethenyl-6-methylpyrazine	1495	peanut, cacao-like	salty (55%)	umami (35%) sweet (10%)	RI/MS/O
11	decanal	1502	wax, fatty	salty (45%)	umami (35%) bitter (20%)	RI/MS/O
12	3-(methylthio)propanol	1715	heated onion	salty (55%)	umami (25%) bitter (20%)	RI/MS/O
13	3-hydroxy-2-methyl-4H-pyran-4-one	1964	malty, roasted	salty (45%)	sweet (40%) umami (15%)	RI/MS/O
14	unknown	/	soy sauce	salty (80%)	umami (20%)	/
Odor descriptors associated with sourness						
1	2-methyl butanal	918	spicy, fresh fruit	sour (50%)	salty (30%) umami (20%)	RI/MS/O
2	acetic acid	1419	vinegar	sour (100%)		RI/MS/O
3	propionic acid	1547	rancid	sour (70%)	bitter (15%) salty (15%)	RI/MS/O
4	butanoic acid	1581	sweaty	sour (80%)	bitter (20%)	RI/MS/O
5	3-methylbutanoic acid	1674	sweaty	sour (100%)		RI/MS/O
6	1-methyl-2-pyrrolidinone	1679	sweaty	sour (85%)	bitter (15%)	RI/MS/O
7	3-methylvaleric acid	1800	sweaty	sour (100%)		RI/MS/O
8	4-methylvaleric acid	1808	rancid	sour (80%)	bitter (20%)	RI/MS/O
9	γ-nonanolactone	2031	coconut-like	sour (70%)	bitter (30%)	RI/MS/O
10	octanoic acid	2067	sweaty	sour (80%)	bitter (20%)	RI/MS/O
Odor descriptors associated with sweetness						
1	2,3-butanedione	969	caramel, butter	sweet (60%)	sour (40%)	RI/MS/O
2	3-methyl-1-butanol	1221	apple-like, brandy-like	sweet (40%)	sour (20%) bitter (20%) umami (20%)	RI/MS/O
3	3-hydroxy-2-butanone	1289	creamy	sweet (45%)	salty (30%) umami (25%)	RI/MS/O
4	2,3-dimethylpyrazine	1344	rice-like, butter	sweet (65%)	salty (10%) umami (25%)	RI/MS/O
5	1-(2-furanyl)-ethanone	1511	milk-like	sweet (80%)	umami (20%)	RI/MS/O
6	benzeneacetaldehyde	1649	honey	sweet (75%)	umami (25%)	RI/MS/O
7	dihydro-5-ethyl-2(3H)-furanone	1703	caramel	sweet (50%)	bitter (30%) umami (20%)	RI/MS/O
8	trimethyl cyclohexadienyl butenone	1819	honey, fruity	sweet (85%)	salty (10%) bitter (5%)	RI/MS/O
9	2-hydroxy-1-methylcyclopenten-3-one	1828	grass-like	sweet (85%)	bitter (15%)	RI/MS/O
10	3-methyl-1,2-cyclopentanedione	1839	caramel, herbal	sweet (65%)	bitter (35%)	RI/MS/O
11	2-methoxyphenol	1861	smoky	sweet (85%)	bitter (15%)	RI/MS/O
12	benzyl alcohol	1880	sweet, floral	sweet (60%)	salty (20%) sour (20%)	RI/MS/O
13	phenethyl alcohol	1910	floral	sweet (85%)	sour (15%)	RI/MS/O
14	2-phenyl-2-butenal	1907	floral	sweet (60%)	salty (40%)	RI/MS/O
15	5-methyl-2-phenyl-2-hexenal	2076	butter, nutty	sweet (60%)	salty (30%) sour (10%)	RI/MS/O
16	4-allyl-2-methoxyphenol	2168	herbal, sweet	sweet (55%)	bitter (30%) salty (15%)	RI/MS/O
	Odor descriptors associated with bitterness					
1	2,3,5-trimethylpyrazine	1416	musty, bitterness	bitter (65%)	sour (35%)	RI/MS/O
2	2,6-dimethoxyphenol	2265	bitterness, green	bitter (90%)	umami (10%)	RI/MS/O
3	2,3-dihydro-3,5-dihydroxy-6-methyl-4(H)-pyran-4-one	2223	herbal	bitter (40%)	sweet (20%) salty (20%) umami (20%)	RI/MS/O
4	benzoic acid	2458	spicy	bitter (40%)	sweet (30%) salty (20%) umami (10%)	RI/MS/O
Odor descriptors associated with umami						
1	citronellol	1766	woody	umami (40%)	bitter (30%) sour (30%)	RI/MS/O

^a^ Retention index. ^b^ Detection frequency (%): the number of panelists that perceived a particular odorant was the denominator, and the number of panelists that could perceive the odorant associated with a certain taste was the mumerator. ^c^ Mode of identification: RI, retention index published in the literature data; MS, mass spectrum verified by comparison with mass spectra database (NIST 14.0); O, odor descriptions of standard or published.

**Table 2:** Sensory evaluation of saltiness for salt solution with and without odorant (paired comparisontest).

Odorant	Odorant Concentration (ppb)	Number of Evaluations with Nose Clip (*n* = 24)		Significance	Number of Evaluations without Nose Clip (*n* = 24)		Significance
		Salt Solution without Odorant	Salt Solution with Odorant		Salt Solution without Odorant	Salt Solution with Odorant	
2-ethyl-3,5-dimethyl pyrazine	0.2	15	9	NS	9	15	NS
	2	17	7	*	3	21	**
	20	22	2	**	1	23	**
2,5-dimethyl pyrazine	1	16	8	NS	13	11	NS
	10	13	11	NS	6	18	*
	100	22	2	**	7	17	*
2,6-dimethyl pyrazine	2	16	8	NS	10	14	NS
	20	14	10	NS	12	12	NS
	200	23	1	**	18	6	*
2-ethyl-5(6)-methyl pyrazine	1	16	8	NS	13	11	NS
	10	17	7	*	12	12	NS
	100	18	6	*	11	13	NS
dimethyl trisulfide	0.025	18	6	*	12	12	NS
	0.25	16	8	NS	7	17	*
	2.5	11	13	NS	11	13	NS
3-(methylthio) propanol	2.5	14	10	NS	14	10	NS
	25	13	11	NS	11	13	NS
	250	17	7	*	6	18	*
tetramethyl pyrazine	10	15	9	NS	11	13	NS
	100	12	12	NS	15	9	NS
	1000	14	10	NS	9	15	NS
3-(methylthio) propanal	0.2	8	16	NS	11	13	NS
	2	9	15	NS	4	20	**
	20	6	18	*	7	17	*

NS: no statistically significant difference between samples, *p* > 0.05 (*n* < 17); * Significantly different at *p* ≤ 0.05 (*n* ≥ 17); ** Significantly different at *p* ≤ 0.01 (*n* ≥ 19) (ISO 5495:2005).

## Data Availability

Data are contained within the article.

## Abbreviations

AEDA	Aroma extract dilution analysis
GC-MS	Gas chromatography−mass spectrometry
2-AFC	2-Alternative forced-choice
GC-O/AT	Gas chromatography-olfactometry/associated taste
OISE	Odor-induced saltiness enhancement
SAFE	Solvent-assisted flavor extraction
RI	Retention index
DF	Detection frequency
MDC	Minimum detection concentration
MC	Middle concentration
